# Clinical Features and Patient Outcomes in Infective Endocarditis with Surgical Indication: A Single-Centre Experience

**DOI:** 10.3390/jcdd11050138

**Published:** 2024-04-29

**Authors:** Fausto Pizzino, Umberto Paradossi, Giancarlo Trimarchi, Giovanni Benedetti, Federica Marchi, Sara Chiappino, Mattia Conti, Gianluca Di Bella, Michele Murzi, Silvia Di Sibio, Giovanni Concistrè, Giacomo Bianchi, Marco Solinas

**Affiliations:** 1 Cardiology Unit, Heart Centre, Fondazione Gabriele Monasterio—Regione Toscana, 54100 Massa, Italy; fpizzino@ftgm.it (F.P.); uparadossi@ftgm.it (U.P.); giovanni.benedetti@ftgm.it (G.B.); federica.marchi@ftgm.it (F.M.); schiappino@ftgm.it (S.C.); 2 Department of Clinical and Experimental Medicine, University of Messina, 98100 Messina, Italy; giancarlo.trimarchi18@gmail.com (G.T.); gianluca.dibella@unime.it (G.D.B.); 3 Department of Surgical Molecular Medical and Critical Area Pathology, University of Pisa, 56124 Pisa, Italy; mattia.contimedchir@gmail.com; 4 Division of Adult Cardiac Surgery, Fondazione Toscana Gabriele Monasterio, 54100 Massa, Italy; disibio@ftgm.it (S.D.S.); concistr@ftgm.it (G.C.); gbianchi@ftgm.it (G.B.); solinas@ftgm.it (M.S.)

**Keywords:** infective endocarditis, heart failure, embolic events, cardiac surgery

## Abstract

Background: Infective endocarditis (IE) is marked by a heightened risk of embolic events (EEs), uncontrolled infection, or heart failure (HF). Methods: Patients with IE and surgical indication were enrolled from October 2015 to December 2018. The primary endpoint consisted of a composite of major adverse events (MAEs) including all-cause death, hospitalizations, and IE relapses. The secondary endpoint was all-cause death. Results: A total of 102 patients (66 ± 14 years) were enrolled: 50% with IE on prosthesis, 33% with IE-associated heart failure (IE-aHF), and 38.2% with EEs. IE-aHF and EEs were independently associated with MAEs (HR 1.9, 95% CI 1.1–3.4, *p* = 0.03 and HR 2.1, 95% CI 1.2–3.6, *p* = 0.01, respectively) and Kaplan–Meier survival curves confirmed a strong difference in MAE-free survival of patients with EEs and IE-aHF (*p* < 0.01 for both). IE-aHF (HR 4.3, 95% CI 1.4–13, *p* < 0.01), CRP at admission (HR 5.6, 95% CI 1.4–22.2, *p* = 0.01), LVEF (HR 0.9, 95% CI 0.9–1, *p* < 0.05), abscess (HR 3.5, 95% CI 1.2–10.6, *p* < 0.05), and prosthetic detachment (HR 4.6, 95% CI 1.5–14.1, *p* < 0.01) were independently associated with the all-cause death endpoint. Conclusions: IE-aHF and EEs were independently associated with MAEs. IE-aHF was also independently associated with the secondary endpoint.

## 1. Introduction

Infective endocarditis (IE) represents a severe clinical syndrome often characterized by a high risk of embolization, uncontrolled infection, or heart failure (HF) [1]. In these high-risk populations, cardiac surgery is indicated to improve survival and symptoms [2]. Although there are risks associated with surgery for these patients, the latest research indicates that undergoing surgical treatment could potentially increase survival rates by as much as 20% within the first year [3,4].

The scope of surgical intervention in infective endocarditis (IE) is broadening, as supported by both American and European guidelines for managing complicated cases [5,6]. With the changing global epidemiology, more patients from the developing world are presenting with IE, leading to a rise in related surgical procedures. This trend means that patients are older and have more comorbidities upon presentation, including a higher prevalence of implanted devices like pacemakers and defibrillators, adding complexity to the management of the condition [7].

Recently, studies showed an increase in surgeries for IE over the past twenty years. According to the ESC-EORP EURO-ENDO study, over 50% of IE patients in tertiary centers underwent surgery [8]. Yet, the rates of surgical treatment differ widely, ranging from 20% to 70%, influenced by the country and the availability of cardiac surgical services, among various factors [9]. This indicates a significant variation in the management of IE across different regions. The high complexity of IE patients demands a comprehensive, multidisciplinary strategy for effective management, as emphasized in current guidelines, which also recognize the potential benefit of early surgical intervention [5,6]. Achieving favorable surgical and long-term outcomes hinges on several factors, including the choice of surgical procedure, operational nuances, and the healthcare center’s expertise. Surgical options are advised for patients experiencing HF, paravalvular abscess, systemic embolization, prosthetic valve infections, persistent sepsis, or sizable vegetations, to optimize treatment outcomes [10]. However, patients with IE represent an heterogenous population ranging from young subjects with few comorbidities to older and more complex patients. Effectively managing IE involves quickly diagnosing the condition, promptly starting antimicrobial treatment, and making decisions about potential complications such as the risk of embolism and the need for high-risk surgery, as well as the optimal timing for such procedures [11,12]. In this context, defining the clinical, laboratory, and imaging characteristics of IE patients with indication for cardiac surgery and their impact on prognosis is of paramount importance. In this retrospective, single-center study, we aimed to identify the prognostic features of patients with IE referring for surgery to our Center from the geographic area of North-West Tuscany.

## 2. Materials and Methods

We retrospectively reviewed the electronic charts of patients referring to our Center for IE with indication for cardiac surgery from October 2015 to December 2018. IE diagnosis was established according to European Society of Cardiology modified criteria for the diagnosis of IE [6]. All patients had a diagnosis of IE in our Center or in other hospitals located in the area. We recorded a set of clinical, laboratory, and imaging features. Clinical features included age; gender; diabetes; chronic kidney disease [(CKD), defined as a glomerular filtration rate < 60 mL/m^2^]; embolic events (EEs); injective drugs addiction; atrial fibrillation (AF); current or recent (within previous 6 months) immunosuppressive therapies (ITs); IE-related acute heart failure (IE-aHF), defined as the rapid onset of heart failure symptoms with an elevation of natriuretic peptides and an urgent need of intensive medical support (diuretics, O_2_-therapy, amine infusion, and mechanical circulatory support) directly caused by IE complications; IE on prosthesis; and the number of days between fever/symptoms onset and the diagnosis of IE. Laboratory features included white blood cell count (WBCC); C reactive protein (CRP); and procalcitonin, measured both as admission value and as peak value. Imaging features included left ventricular ejection fraction (LVEF); vegetations; abscess; intracardiac fistula; leaflet perforation; pseudoaneurysm; and prosthetic detachment.

The study protocol is in line with the Helsinki Declaration and was approved by the Institutional Review Board of Fondazione Toscana “Gabriele Monasterio”, Massa, Italy (approval number 11853; approved 28 March 2018).

Follow-up was carried out through telephonic interviews, by consulting official public registries, and by means of outpatient evaluations. Our primary endpoint consisted of a composite of major adverse events (MAEs) includes all-cause death, re-hospitalizations (readmission for cardiovascular causes other than infective ones), and IE relapses. The secondary endpoint was all-cause death. We used a commercial machine equipped with a phased array 1–5 mHz cardiac probe and with a 2–7 MHz transesophageal probe (IE33 Philips, Amsterdam, The Netherlands). All patients underwent both transthoracic and transesophageal echocardiography. LVEF was computed with the modified biplane Simpson’s method. Abscess was defined as the presence of an abnormal echolucent/echodense inhomogeneous area located in the peri-annular region or in the mitral-aortic continuity. Pseudoaneurysm was defined as an abnormal, pulsatile, echo-free space, with the presence of blood flow at Color-Doppler. Fistula was identified when an abnormal communication between two different cardiac chambers was present with Color-Doppler flow evidence. Leaflet perforations were identified as an interruption of endocardial tissue continuity traversed by Color-Doppler flow. Vegetations were defined as filamentous, pedunculated masses attached to native or prosthetic leaflets/rings [13]. Prosthetic detachment was defined as a de novo identification of periprosthetic leak with or without prosthesis rocking [14]. All patients at admission underwent blood sampling for the dosage of CRP, procalcitonin, and blood count including WBCC. The measurement of CRP and procalcitonin was performed using commercial kits.

Continuous variables were expressed as means ± SD or median (25th; 75th percentiles) as appropriate. Categorical variables were expressed as numbers and percentages. The comparison between continuous variables was performed using Student’s independent samples *t*-test or Wilcoxon test, according to distribution. The comparison between categorical variables was performed either using the Chi-square test or Fisher’s exact test if an expected cell count was less than 5. In the primary analysis, univariate Cox regression analysis was used to investigate the association of every single variable with the events. Subsequently, the variables showing a significant association with the events were included in a multivariate analysis to define the independence. Regarding the secondary endpoint, the proportional hazard rule was violated, and consequently, we used logistic regression analysis to investigate the association between all-cause death and variables. Because of the low incidence of events, we performed only an adjustment for age in the bivariate model. We used receiver operating characteristic curves analysis and the Youden criterion to identify the sensitivity, specificity, and cut-offs of the variables associated with the endpoint. Kaplan–Meier curves and Log-Rank test were used to analyze endpoint-free survival. All tests were performed as two-sided. Statistical significance was considered for a *p* value < 0.05. Statistical software used were SPSS version 23 (IBM Corp. 2015. Armonk, NY, USA, 2015) and MedCalc version 14.8.1 (MedCalc Software bvba, Ostend, Belgium, 2014).

## 3. Results

In the considered time interval, we identified 139 patients admitted for IE with indication for surgery. Among these, 15 patients were excluded because of incomplete data; 16 patients were lost to follow-up, while 6 patients were excluded because the surgical indication was not confirmed. Therefore, we included 102 patients in the final analysis. The features of the population are summarized in Table 1 and Table 2.

About half of the patients had IE involving a prosthesis. A total of 38.2% of patients showed EE, and the most frequent localizations were the brain and spleen (both 38.46%), followed by the lungs (12.82%) and inferior limbs (5.13%). One third of the patients presented with IE-aHF, while only 13.7% had injective drug addiction. The days from symptom onset to IE diagnosis were widely variable, with a median of 20 days (7–50). Vegetations were the most frequent echocardiographic finding and were identified in 83.3% of the population, followed by abscess (29.4%) and leaflet perforation (25.5%). Mean LVEF was 55% and in most cases, it fell in the normal range. Patients presenting with IE on prosthesis had a median prosthesis life of 6 months from implantation. WBC, CRP, and procalcitonin at admission were elevated in most patients. The most frequently isolated microorganism was Staphylococcus Epidermidis (SE, 22%), followed by Staphyloccocus Aureus (SA, 14%) and Enterecoccus Faecalis (EF, 14%). Regarding annual incidence per 10,000 inhabitants, in 2016, it appeared lower (1.56 per 100,000 inhabitants), while in 2017, we observed an increase of the incidence, which was confirmed in 2018 (2.74 and 2.75, respectively, per 10,000 inhabitants). In 57 cases, aortic valve replacement was performed (46 biological and 11 mechanical); in 43 cases, a mitral valve surgery was performed (18 biological prostheses, 12 mechanical prostheses, and 13 repairs); and 7 patients had tricuspid valve surgery (4 biological prostheses and 3 repairs). In 10 patients, surgical indication was not confirmed because of extremely high surgical risk; then, only medical therapy was performed. Eight patients had double aortic-mitral replacement (five biological and three mechanical); one patient had mitral-tricuspid replacement with biological prosthesis, and five patients underwent combined aortic biological replacement and mitral repair while two patients had mechanical mitral replacement and tricuspid repair. Patients who did not undergo intervention had a trend to have more MAEs (16% vs. 3.8% *p* = 0.05) and died significantly more (27.8% vs. 6%, *p* = 0.01) than patients in whom indication for surgery was confirmed.

For the primary composite endpoint, the median follow-up was 276 days (IQR: 75–483). A total of 18 Patients died; 24 patients had a hospitalization and 8 patients had IE relapse. Therefore, our composite endpoint counted 50 MAEs. For the secondary endpoint of death, the median follow-up was 328 days (86–587).

Sorting the population according to the occurrence of MAEs, we observed that patients with MAEs were older (69 ± 12 years vs. 63.7 ± 16 years, *p* < 0.05) and with a higher prevalence of IE-aHF (48 vs. 17.3%, *p* = 0.001). Patients with MAEs had a shorter time-interval between symptom onset and diagnosis [15 days (6–30) vs. 30 (7–60), *p* = 0.03], probably because of the more severe and rapid evolution of the disease. It is worth noting that, in our population, time between symptoms and diagnosis represents a sort of indicator of the timing from symptoms to surgery since all patients underwent intervention within 2 weeks from diagnosis. Patients with MAEs had an EE in 50% of cases while patients without MAEs only in 26.9% of cases (*p* = 0.02). It is worth noting that 50% of our population had IE on prosthesis, but the prevalence in the two groups of patients was not significantly different despite a trend to be lower in the no-MAE group in comparison to the MAE group (42.3% vs. 58%, *p* = 0.1). None of the laboratory data were significantly different in the two groups. Among imaging features, abscess (40% vs. 19.2%, *p* = 0.02) and prosthetic detachment (32% vs. 15.4%) were more frequent in the MAE group.

Regarding the secondary endpoint, patients who had died at follow-up were older (73.3 ± 9 vs. 64.6 ± 15, *p* < 0.01) and presented a higher frequency of IE-aHF at presentation, and higher CRP at admission [8 (3.9–16) vs. 3.8 (1.4–7.6), *p* = 0.02] and higher peak of PCT [2 (0–8) vs. 0.1 (0–2), *p* = 0.01] were higher in the same group. Among imaging features, LVEF was lower in patients in the death-group (48 ± 16 vs. 56 ± 9, *p* < 0.05), and at the same time, abscess (50% vs. 25%, *p* = 0.03) and prosthetic detachment (50% vs. 17.9%, *p* < 0.01) were more prevalent. All indicators demonstrated an independent association with all-cause death when adjusted for age (Table 3).

To demonstrate association with MAEs, we included the variables showing significant differences among groups in a univariate Cox regression model. We found that, among clinical features, EEs (HR: 2, 95% CI: 1.1–3.4, *p* = 0.02) and IE-aHF (HR: 2.1, 95% CI: 1.2–3.7, *p* = 0.01) were associated with MAEs. Regarding imaging features, only the finding of abscess (HR: 2.1, 95% CI: 1.2–3.8, *p* < 0.01) was associated with the occurrence of the primary endpoint at follow-up (Table 4).

**Figure 1:**
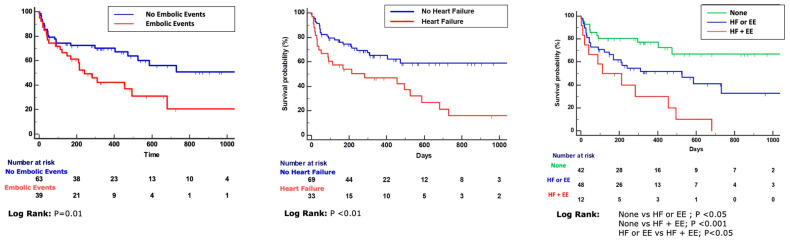
Kaplan–Meier survival curves showing the different survival of patients affected by infective endocarditis, on the left, with and without embolic events (EEs), in the center with and without heart failure (HF), and on the right, with EE and HF, without EE and HF, or with one of the above-mentioned clinical features.

Aiming to identify the variables independently associated with MAEs, we included the variables in a multivariate model. We observed that only the presence of IE-aHF and EEs was independently associated with MAEs (HR 1.9, 95% CI 1.1–3.4, *p* = 0.03, and HR 2.1, 95% CI 1.2–3.6, *p* = 0.01, respectively). Kaplan–Meier survival curves confirmed a strong difference in the MAE-free survival of patients with EEs and IE-aHF (*p* < 0.01 for both). Moreover, clustering together patients with both IE-aHF and EEs, we found significant differences in survival vs. patients with isolated IE-aHF or EEs and vs. patients with neither IE-aHF nor EEs (Figure 1).

Analyzing separately the incidence of the three risk factors associated with poor prognosis according to the three different endpoints (Table 5), it emerges that IE-aHF appears as the main driver of death. Indeed, patients who had died at follow-up had a 66.7% incidence of IE-aHF, and 80% of patients with IE-aHF had died at follow-up. The incidence of EEs tended to be particularly high in patients who experienced re-hospitalization as well as patients with endocarditis relapse, while patients with abscess tend to have mainly death and re-hospitalization at follow-up.

**Figure 2:**
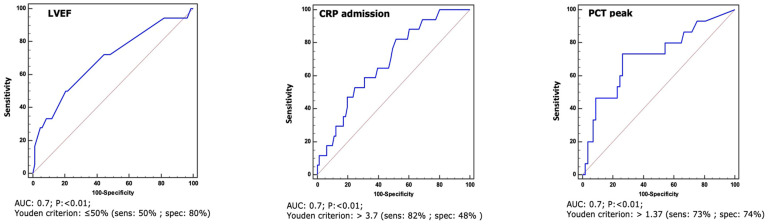
ROC curves with sensitivity and specificity for a cut-off of LVEF ≤ 50%, CRP at admission > 3.7 mg/dL, and PCT peak > 1.37 µg/L, referring to the secondary endpoint of all-cause death.

Regarding the secondary endpoint of death from any cause, significant differences between groups emerged for the following variables: IE-aHF, CRP at admission, procalcitonin peak, LVEF, abscess, and prosthetic detachment. All variables were found to be associated with the secondar endpoint in the univariate logistic regression analysis. After adjustment for age, all variables, except for procalcitonin peak, remained independently associated with death. A ROC curve analysis revealed that the best cut-offs for continuous variables associated with the endpoint were LVEF ≤ 50%, CRP at admission > 3.7 mg/dL, and PCT peak > 1.37 µg/L (Figure 2).

## 4. Discussion

In the era of interventional cardiology and cardiothoracic surgery, IE represents a critical variable and a serious public health issue since the presence of intracardiac implants is a common risk factor. In the case of uncontrolled infection, elevated embolic risk, or HF, cardiac surgery is the treatment of choice in combination with empiric and targeted antimicrobial therapy [15]. Before antibiotics, infective endocarditis (IE) often led to fatal outcomes. While antibiotics provided a cure in certain cases, mortality rates have continued to be elevated, primarily attributed to heart failure resulting from valve damage [16]. The application of surgical intervention has significantly improved the prognosis, initially as the next step after antibiotic therapy, but now employed even during active disease episodes [17]. Observational studies have demonstrated the efficacy of surgery for patients with active IE experiencing complications such as heart failure or uncontrolled infection [18,19].

This concept has been further underlined by the latest 2023 guidelines for the management of endocarditis of the European Society of Cardiology, which highlight the crucial role of cardiac surgery in directly addressing the infection’s source, in eliminating any embolic hazards, and in reducing heart failure risks [6]. In our study, only patients with IE with indication for surgery were enrolled and two endpoints were investigated: a primary endpoint, consisting of a composite of major adverse events (MAEs) including all-cause death, hospitalizations, and IE relapses, and a secondary endpoint for all-cause death. According to our data, patients with MAEs were older and with a higher prevalence of IE-aHF, embolic events, injective drug addiction, and prosthetic detachment. This finding is confirmed in the latest ESC 2023 guidelines that report older age, heart failure, cerebral complications, and peri-annular complications as strong predictors of adverse outcomes in patients with infective endocarditis [6]. As underlined by Bea et al., the management of IE in elderly patients is very challenging due to the atypical presentation of the disease, comorbidities like diabetes and cancer, more frequent enterococcal etiology, and less suitability for surgical intervention [20]. Also, Van den Brink et al. found that older age is an independent prognostic factor for mortality in patients affected by IE for the same reasons as those listed before [21].

Numerous studies have highlighted heart failure’s pivotal role as a significant prognostic factor in endocarditis, emphasizing its critical influence on patient outcomes and disease progression [22,23]. In our own investigation, we defined heart failure in infective endocarditis as the rapid onset of heart failure symptoms, accompanied by elevated levels of natriuretic peptides and an urgent requirement for intensive medical support, resulting directly from complications of infective endocarditis. Furthermore, data from the EURO-ENDO registry support this conclusion, emphasizing that congestive heart failure independently predicts mortality in patients with IE [8].

Heart failure in endocarditis is more commonly a result of valve dysfunction, specifically valve failure. Consequently, addressing this valve issue through valve replacement or repair would likely resolve the problem without aggravating the prognosis for urgent surgery [11,24,25].

According to our data, IE-aHF represents an independent negative prognostic factor that significantly reduces MAE-free survival. This finding agrees with what is reported by Nadji et al., who enrolled two hundred and fifty-nine consecutive patients with definite left-sided native valve IE and established that new-onset congestive heart failure was independently predictive of in-hospital and 1-year mortality, while early surgery was independently associated with improved 1-year survival [23]. Our sample, unlike the one selected by Nadji et al., was composed of both patients with endocarditis on the native valve and on the prosthetic valve, underlining the negative prognostic significance of IE-aHF which is also valid for a more heterogeneous population and regardless of the type of valve (native or prosthetic) involved in the disease. Another aspect of IE regards embolic events (EEs), which are frequent and life-threatening complications in IE; up to 25% of IE patients exhibit EEs at the time of diagnosis [8]. In our study, embolic events (EEs) were independently associated with major adverse events and had a negative prognostic value on patients’ MAE-free survival. The definition of EEs used in our study also includes cerebral embolism, which can lead to serious complications like neurological sequalae that occur in 25% to 70% of cases [26]. There is a noteworthy inverse relationship between the prognosis of infective endocarditis and moderate to severe ischemic stroke and brain hemorrhage [27,28].

Furthermore, when we clustered together patients with both IE-aHF and EEs, we discovered substantial differences in survival between patients who had neither IE-aHF nor EE, as well as between patients who had both or only one of the two. This demonstrates that the coexistence of these two clinical conditions worsens the prognosis of patients with IE. In our study, abscess and prosthetic detachment were found to be independent predictors for death from any cause. According to Weber et al., individuals with perivalvular abscesses had much higher 30-day mortality and postoperative sequelae [29]. Similar findings were obtained by Straw et al., who reported that patients with intra-cardiac abscess complicating their IE experience have adverse outcomes, especially when they do not undergo surgery [30]. In our study, baseline C-reactive protein was found to be independently associated with all cause-death with a cut-off 37 mg/L. This result is in concordance with that of Mohanan et al., who demonstrated that a CRP > 40 mg/L predicted adverse outcomes with a sensitivity of 73% and a specificity of 99% [31].

## 5. Limitations

The present study has several limitations. First, the retrospective design of the study decreases the statistical power and the consequent conclusions. Second, the number of patients was relatively low; however, the numerical population is acceptable considering the single-center recruitment and the relatively short time window of the study. Third, the examined population is widely heterogenous in terms of age, clinical presentation, and treatment; however, this reflects the real-life setting. Fourth, we did not include in the analysis the antimicrobial treatment because of the large fragmentary data regarding mainly doses and treatment duration; this could be a confounding feature. Fifth, the secondary endpoint of all-cause death counted only 18 events, and thus we could only adjust the association for age. Sixth, patients with IE on prosthesis or with prosthetic detachment may suffer from a selection bias since their operatory risk was superior considering that they underwent a repeat intervention; however, no association with MAEs was observed for patients with IE on prosthesis, and prosthetic detachment was only associated with all-cause death.

## 6. Conclusions

The study confirms that patients with IE with an indication for cardiothoracic surgery represents a high-risk population with an elevated incidence of adverse events in the mid- and long term. The presence of IE-aHF and the occurrence of peripheral septic embolization emerged as features independently associated with the composite primary endpoint. IE-aHF was also associated with the secondary endpoint of all-cause death as alongside the levels of CRP at admission, procalcitonin peak, LVEF, the presence of abscess, and prosthetic detachment. The results highlight the importance to identify specific features associated with poor prognosis in this heterogeneous setting of high-risk patients.

## Figures and Tables

**Table 1:** Clinical, laboratory, and imaging features of patients affected by infective endocarditis, further subdivided on the basis of the primary endpoint.

	Whole Population (102)		Primary Endpoint				
Clinical Features			No MAE (52)		MAE (50)		*p*
Age	66	±14	63	±16	69	±12	<0.05
Male gender	72	71%	38	73.1%	34	68%	0.6
Diabetes	14	13.7%	6	11.5%	8	16%	0.5
CKD	30	29.4%	14	26.9%	16	32%	0.6
Embolic events	39	38.2%	14	26.9%	25	50%	0.02
Injective drug addiction	14	13.7%	11	21.2%	3	6%	0.03
Atrial fibrillation	22	21.6%	9	17.3%	13	26%	0.3
IT	7	6.9%	3	5.8%	4	8%	0.6
IE-aHF	33	32.4%	9	17.3%	24	48%	0.001
IE on prosthesis	51	50%	22	42.3%	29	58%	0.1
Days S-D	20	(7–50)	30	(7–60)	15	(6–30)	0.03
Laboratory data							
WBCC admission (×10^3^/µL)	14	(9–28)	11	(8–26)	15	(9–28)	0.1
CRP admission (mg/dL)	4	(1.5–9.7)	4	(1.1–17.2)	4	(1.8–9.7)	0.2
PCT admission (µg/L)	0.13	(0–0.78)	0.17	(0–0.50)	0.12	(0–0.78)	0.7
WBCC peak (×10^3^/µL)	19	(14–62)	17	(13–30)	24	(15–35)	0.1
CRP peak (mg/dL)	10.6	(4.2–17.5)	10.8	(4.5–17.2)	10.4	(3.9–16)	0.9
PCT peak (µg/L)	0.60	(0.14–3.2)	0.63	(0.14–3.2)	0.5	(0.1–3)	0.5
Imaging Features							
LVEF (%)	55	±11	56	±10	53	±12	0.2
Vegetation	85	83.3%	43	82.7%	42	84%	0.8
Abscess	30	29.4%	10	19.2%	20	40%	0.02
Fistula	3	2.9%	3	5.8%	0	0.0%	0.09
Leaflet perforation	26	25.5%	17	32.7%	9	18%	0.09
Pseudoaneurysm	10	9.8%	7	13.5%	3	6%	0.2
Prosthetic detachment	24	23.5%	8	15.4%	16	32%	<0.05

CKD: chronic kidney disease; IT: immunosuppressive treatment; IE: infective endocarditis; IE-aHF: infective endocarditis-related heart failure; S-D: symptoms to diagnosis; WBCC: white blood cell count; CRP: C-reactive protein; PCT: procalcitonin; LVEF: left ventricular ejection fraction.

**Table 2:** Clinical, laboratory, and imaging features of patients affected by infective endocarditis, further subdivided on the basis of the secondary endpoint.

	Whole Population (102)		Secondary Endpoint				
Clinical Features			No Death (84)		Death (18)		*p*
Age	66	±14	64.6	±15	73.3	±9	<0.01
Male gender	72	71%	60	71.4%	12	66.7%	0.7
Diabetes	14	13.7%	13	15.5	1	5.6%	0.3
CKD	30	29.4%	22	26.2%	8	44.4%	0.1
Embolic events	39	38.2%	30	35.7%	9	50%	0.3
Injective drug addiction	14	13.7%	13	15.5%	1	5.6	0.5
Atrial fibrillation	22	21.6%	17	20.2%	5	27.8%	0.5
IT	7	6.9%	6	7.1%	1	5.6%	0.9
IE-aHF	33	32.4%	22	26.2%	11	61.1%	<0.01
IE on prosthesis	51	50%	39	46.4%	12	66.7%	0.1
Days S-D	20	(7–50)	21	7–60	7	5–20	0.07
Laboratory data							
WBCC admission (×10^3^/µL)	14	(9–28)	13.9	8.5–18	14	9–17	0.8
CRP admission (mg/dL)	4	(1.5–9.7)	3.8	1.4–7.6	8	3.9–16	0.02
PCT admission (µg/L)	0.13	(0–0.78)	0.1	0–0.5	0.6	0.0–3.6	0.3
WBCC peak (×10^3^/µL)	19	(14–62)	19	15–24	19	14–25	0.5
CRP peak (mg/dL)	10.6	(4.2–17.5)	10.6	1.8–17	13.1	3.9–18.9	0.7
PCT peak (µg/L)	0.60	(0.14–3.2)	0.1	0–2	2	0–8	0.01
Imaging Features							
LVEF (%)	55	±11	56	±9	48	±16	<0.05
Vegetation	85	83.3%	71	84.5%	14	77.8%	0.5
Abscess	30	29.4%	21	25%	9	50%	0.03
Fistula	3	2.9%	3	3.6%	0	0%	0.4
Leaflet perforation	26	25.5%	24	28.6%	2	11.1%	0.1
Pseudoaneurysm	10	9.8%	9	10.7%	1	5.6%	0.7
Prosthetic detachment	24	23.5%	15	17.9%	9	50%	<0.01

CKD: chronic kidney disease; IT: immunosuppressive treatment; IE: infective endocarditis; IE-aHF: infective endocarditis-related heart failure; S-D: symptoms to diagnosis; WBCC: white blood cell count; CRP: C-reactive protein; PCT: procalcitonin; LVEF: left ventricular ejection fraction.

**Table 3:** Univariate and multivariate analysis of clinical features associated with the secondary endpoint of all-cause death, adjusted for age.

LogR	Univariate			Multivariate(Adjusted for Age)		
Variable	HR	(95% CI)	*p*	HR	(95% CI)	*p*
Age	1.1	1–1.1	<0.05			
IE-aHF	4.4	1.5–12.8	<0.01	4.3	1.4–13	<0.01
CRP admission (LG10)	3.6	1.2–11	<0.05	5.6	1.4–22.2	0.01
PCT peak (LG10)	2.7	1.1–6.8	<0.05	2.6	1–6.8	0.05
LVEF	0.9	0.9–1	0.01	0.9	0.9–1	<0.05
Abscess	3	1.1–8.5	<0.05	3.5	1.2–10.6	<0.05
Prosthetic detachment	4.6	1.6–13.5	<0.01	4.6	1.5–14.1	<0.01

IE-aHF: infective endocarditis-related heart failure; CRP: C-reactive protein; PCT: procalcitonin; LVEF: left ventricular ejection fraction.

**Table 4:** Univariate and multivariate analysis of clinical features associated with the primary endpoint of major adverse events (MAEs).

	Univariate			Multivariate		
Variable	HR	(95% CI)	*p*	HR	(95% CI)	*p*
Abscess	2.1	1.2–3.8	0.01	1.8	1.1–3.4	0.06
IE-aHF	2.1	1.2–3.7	0.01	1.9	1.1–3.4	0.03
Embolic events	2	1.1–3.4	0.02	2.1	1.2–3.6	0.01
IE on prosthesis	1.5	0.9–2.6	0,2			
Prosthetic detachment	1.5	0.8–2.9	0.2			
Age	1.02	1–1.1	0.1			
Days S-D	0.9	0.9–1	0.05			
Injective drug addiction	0.4	0.001–1.4	0.2			

IE: infective endocarditis; IE-aHF: infective endocarditis-related heart failure; S-D: symptoms to diagnosis.

**Table 5:** Incidence of the three risk factors associated with poor prognosis according to the three different endpoints.

	MAEs						
	Death (18)		Endocarditis Relapse (8)		Re-Hospitalization (24)		
Variable	No (% within Death)	% within Row	No (% within ER)	% within Row	No (% within Re-H)	% within Row	*p*
Abscess	9 (50%)	45%	2 (25%)	10.0%	9 (37.5%)	45%	NS
IE-aHF	12 (66.7%)	80%	1 (12.5%)	6.7%	2 (8.3%)	13.3%	<0.001 *
EEs	9 (50%)	36%	4 (50%)	16.0%	12 (50%)	48%	NS
*p* (columns)	<0.001 §		NS		NS		

* Death vs. endocarditis relapse and vs. re-hospitalization; § IE-aHF vs. abscess and EE. Abbreviations: IE: infective endocarditis; IE-aHF: infective endocarditis-related heart failure; EEs: embolic events; ER: endocarditis relapse; Re-H: re-hospitalization.

## Data Availability

The data presented in this study are available on request from the corresponding author. The data underlying this article will be shared on reasonable request to the corresponding author.
